# Effect of Expanded Hemodialysis with the Theranova Dialyzer on the Platelet-to-Lymphocyte Ratio and Inflammatory Markers

**DOI:** 10.3390/toxins17110521

**Published:** 2025-10-22

**Authors:** You Hyun Jeon, Hee-Yeon Jung, Ji-Young Choi, Sun-Hee Park, Chan-Duck Kim, Yong-Lim Kim, Jeong-Hoon Lim, Jang-Hee Cho

**Affiliations:** Division of Nephrology, Department of Internal Medicine, Kyungpook National University Hospital, School of Medicine, Kyungpook National University, Daegu 41944, Republic of Korea; yh-jeon@knu.ac.kr (Y.H.J.); hy-jung@knu.ac.kr (H.-Y.J.); jyss1002@hanmail.net (J.-Y.C.); sh-park@knu.ac.kr (S.-H.P.); drcdkim@knu.ac.kr (C.-D.K.); ylkim@knu.ac.kr (Y.-L.K.)

**Keywords:** expanded hemodialysis, hemodialysis, high-flux, inflammation, medium cut-off membrane

## Abstract

The platelet-to-lymphocyte ratio (PLR) has been used as a marker of inflammation, endothelial damage, and a predictor of mortality. Expanded hemodialysis (HDx) using medium cut-off dialyzer (MCO) can effectively clear medium-sized uremic toxins. This study evaluated the effect of the Theranova dialyzer, a type of MCO dialyzer, on PLR and uremia-related inflammatory markers. A total of 44 patients with maintenance hemodialysis (HD) using high-flux dialyzer were randomly allocated to the Theranova or high-flux group. PLR and inflammatory markers including fibroblast growth factor 23, tumor necrosis factor-α (TNF-α), and interleukin 6 were evaluated every 4 weeks. The changes in PLR and the reduction ratio of inflammatory markers were compared between two groups during the 12-week study period. The baseline characteristics and PLR were not different between groups. After 12 weeks, the levels of PLR, and TNF-α were significantly lower in the Theranova group compared to the high-flux group (all *p* < 0.05). The generalized estimating equation model also revealed a significant decrease in PLR over time in the Theranova group than in the high-flux group (*p* = 0.04). The fold change in 12-week PLR to baseline PLR was lower in the Theranova group than in the high-flux group (*p* = 0.03). In the multivariable linear regression analysis, the Theranova dialyzer showed a negative correlation with the PLR fold change (β = −0.32, *p* = 0.04). Our results showed that HDx using the Theranova dialyzer improves PLR over time compared to the high-flux HD. The superior removal of the inflammatory uremic toxins by the Theranova dialyzer may have reduced inflammation and inflammation-related complications in HD patients.

## 1. Introduction

Inflammation plays a crucial role in the development and progression of kidney disease [1]. Even after the initiation of hemodialysis (HD), patients with end-stage kidney disease (ESKD) continue to experience persistent inflammation, due to the accumulation of uremic toxins, oxidative stress, and repeated exposure to bioincompatible dialysis membranes [2]. This ongoing inflammatory state significantly exacerbates their risk of cardiovascular morbidity and mortality [3], underscoring the need for more effective strategies to manage inflammation in HD patients. The platelet-to-lymphocyte ratio (PLR), a readily available and cost-effective biomarker, is elevated in patients with chronic kidney disease (CKD) [4] and has been identified as a surrogate marker for predicting both all-cause and cardiovascular mortality in ESKD patients [5].

As kidney function deteriorates in CKD patients, uremic toxins accumulate, including various proinflammatory cytokines and growth factors [6]. These elevated levels of uremic toxins perpetuate chronic inflammation, a primary factor contributing to cardiovascular morbidity and mortality [7,8]. Among them, interleukin 6 (IL-6), tumor necrosis factor-α (TNF-α), and fibroblast growth factor 23 (FGF-23) are robust independent predictors of all-cause mortality in stage 5 CKD patients, with IL-6 also serving as a reliable classifier of clinically overt cardiovascular disease [9,10].

Uremic solutes vary in molecular weight and exhibit different removal patterns during dialysis [6]. Middle-molecular uremic toxins are notably challenging to eliminate through conventional HD, which primarily relies on diffusion [11]. However, advancements in dialysis technology have facilitated the removal of a broader spectrum of uremic solutes. Recent developments have introduced medium cut-off (MCO) dialyzers, such as the Theranova dialyzer, which features a tailored pore size designed to enhance the clearance of uremic toxins around 50 kDa in molecular weight.

Although prior studies have demonstrated that MCO dialyzers improve the clearance of circulating inflammatory mediators, most investigations have been limited to biochemical markers. The novelty of the present study lies in linking these molecular effects to a simple and routinely available hematologic index, PLR, which is closely associated with patient outcomes.

The application of MCO dialyzers for the removal of proinflammatory substances of middle molecular weight may ameliorate the inflammatory state in HD patients. This study, therefore, investigates the impact of the Theranova dialyzer compared to high-flux dialyzers on reducing the PLR and inflammatory markers.

## 2. Results

### 2.1. Patient Characteristics

**Figure 1:**
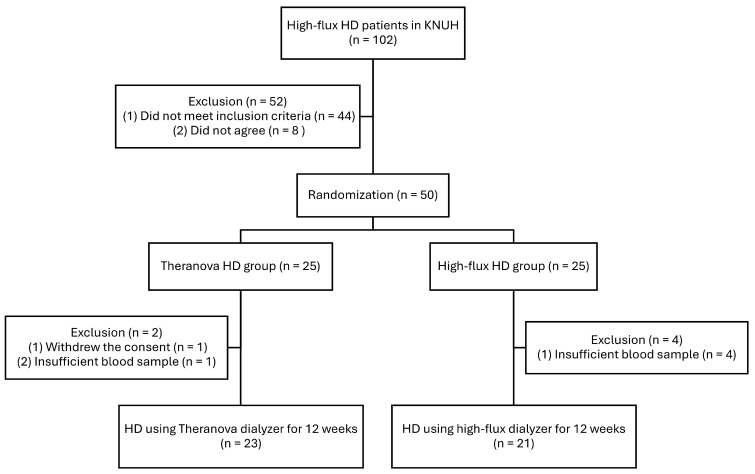
Flow diagram of this study. Abbreviations: HD, hemodialysis; KNUH, Kyungpook National University Hospital.

Among the 50 patients initially randomized, 44 eligible patients were included in the final analysis (Figure 1). Table 1 delineates the baseline characteristics of patients in the Theranova and High-flux groups. Demographics, laboratory results, and dialysis prescriptions were comparable across both groups.

### 2.2. Comparison of Changes in Inflammation-Related Markers

**Figure 2:**
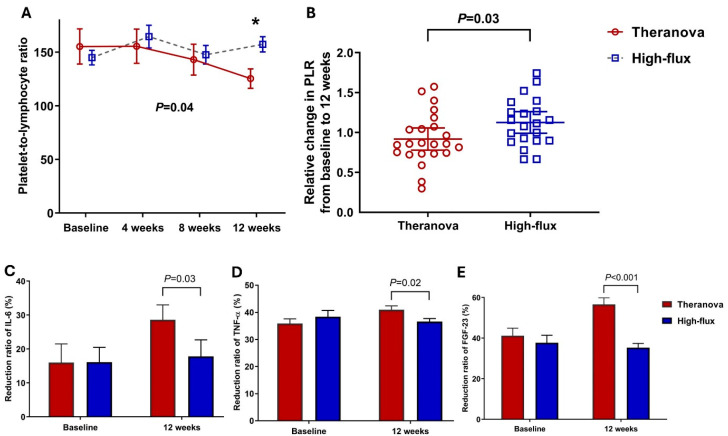
The Theranova group showed an improvement in PLR compared to the high-flux group. (**A**) Serial changes in the PLR using generalized estimating equation models (*p* = 0.04), (**B**) The relative change in the PLR from baseline to 12 weeks (*p* = 0.03). Comparison of reduction ratios in inflammatory markers (**C**) IL-6, (**D**) TNF-α, and (**E**) FGF-23 (all *p* < 0.05) demonstrated the superior efficiency of the Theranova dialyzer. * indicates a significant difference between the two groups at 12 weeks. Abbreviations: PLR, platelet-to-lymphocyte ratio; IL-6, interleukin 6; TNF-α, tumor necrosis factor-alpha; FGF-23, fibroblast growth factor 23.

At baseline, leukocyte and platelet counts, along with levels of inflammation-related markers, did not differ significantly between the groups (Table 2). At 12 weeks, TNF-α levels were markedly lower in the Theranova group compared to the high-flux group (*p* = 0.04). Figure 2A illustrates the results from a generalized estimating equation model that assessed the association between dialyzer type and serial changes in the PLR throughout this study. The generalized estimating equation model revealed a consistent decreasing trend in the PLR in the Theranova group (*p* = 0.04). The relative change in the PLR at 12 weeks compared to baseline was significantly lower in the Theranova group than in the high-flux group (*p* = 0.03, Figure 2B). Comparison of the reduction ratios of inflammatory markers revealed that the Theranova group exhibited significantly higher removal rates of FGF-23, TNF-α, and IL-6 in at 12 weeks (all *p* < 0.05, Figure 2C–E).

### 2.3. Factors Associated with the Reduction in PLR

Multivariable linear regression analysis identified factors associated with the relative change in the PLR from baseline to 12 weeks (Table 3). The analysis indicated an inverse relationship between the use of the Theranova dialyzer and the PLR change (β = −0.32, *p* = 0.04). In contrast, cardiovascular disease showed a positive correlation with the PLR change (β = 0.40, *p* = 0.01).

## 3. Discussion

This study demonstrated that the use of the Theranova dialyzer was associated with a significant reduction in the inflammatory status compared to the high-flux group, as reflected by decreases in the PLR and several inflammatory mediators. These findings indicate that expanded hemodialysis (HDx) with a Theranova dialyzer has the potential to mitigate chronic inflammation in patients receiving maintenance HD.

The PLR serves as an effective marker for ongoing monitoring of inflammatory status due to its ease of measurement and low cost. Extensive research indicates that an elevated PLR is linked with adverse outcomes in patients with ESKD. An increased PLR correlates positively with C-reactive protein levels, reflecting the inflammatory state in HD patients [4]. Moreover, erythropoietin-resistant anemia, which involves chronic inflammation in its pathogenesis, also shows a significant correlation with PLR [12].

The reduction in the PLR observed in the Theranova group can be attributed to the improved removal of harmful middle molecules, including cytokines such as TNF-α and IL-6, as well as growth factors like FGF-23. Chronic exposure to these mediators is well known to contribute to systemic inflammation, endothelial dysfunction, and cardiovascular complications in patients with ESKD [2,3]. By enhancing their clearance, the MCO dialyzer may alleviate the overall inflammatory burden, which is reflected in a lower PLR. Importantly, the PLR is an inexpensive and routinely available parameter that may serve as a practical surrogate marker for monitoring inflammatory status in patients with dialysis.

Our results are consistent with previous prospective randomized trials reporting that MCO dialyzers reduce circulating inflammatory cytokines and oxidative stress more effectively than high-flux dialyzers [13,14,15,16]. Furthermore, when compared with online hemodiafiltration, their effects are generally comparable and, in some cases, even superior, underscoring the potential clinical advantage of MCO membranes in controlling systemic inflammation [17,18,19,20]. However, most prior studies have focused exclusively on biochemical markers. The present analysis extends this knowledge by linking the enhanced removal of inflammatory mediators to hematologic indices, such as the PLR, which are closely associated with patient outcomes [12]. This approach suggests that MCO membranes may influence both molecular and clinical aspects of inflammation.

From a clinical perspective, these results are particularly important. Elevated PLR has been associated with poor survival and erythropoietin resistance in dialysis patients [12]. Previous studies have shown that the use of the Theranova dialyzer was associated with improvements in the erythropoietin resistance index [15,21], and this effect may be related to a concomitant reduction in the PLR. Our findings therefore provide additional evidence supporting this relationship, suggesting that lowering PLR with MCO membranes could translate into improved anemia management and potentially reduced cardiovascular morbidity and mortality. Although the study duration was limited to 12 weeks, the sustained decline in PLR suggests that longer-term use of MCO membranes may yield clinical benefits.

This study has several limitations. The relatively short duration and small sample size prevented the assessment of important clinical outcomes such as hospitalization, cardiovascular events, or mortality. Additionally, the effect of MCO membranes on specific inflammatory mediators varied, and the underlying mechanisms of differential clearance are not yet fully understood. Therefore, future trials with larger cohorts and extended follow-up are needed to validate our findings and clarify which biomarkers are most effectively removed, as well as to establish whether these improvements ultimately translate into tangible benefits for patients.

## 4. Conclusions

In conclusion, HDx with the Theranova dialyzer was associated with reductions in both PLR and inflammatory mediators compared with high-flux HD. These results suggest that the use of MCO membranes may offer an effective approach to reducing chronic inflammation in HD patients, with potential benefits for long-term clinical outcomes.

## 5. Materials and Methods

### 5.1. Patients and Study Design

This study constitutes a post hoc analysis of a randomized controlled trial (RCT) involving maintenance HD patients using MCO and high-flux dialyzers. The original RCT registered 50 adult patients who had been receiving HD for a minimum of three months in Korea. Patients were recruited from Kyungpook National University Hospital, and a total of 102 patients were screened; 52 were excluded (44 did not meet the inclusion criteria and 8 declined to participate). Details on the inclusion and exclusion criteria can be found on the Clinical Research Information Service (CRiS) website (http://cris.nih.go.kr (accessed on 25 July 2018); KCT0003026) and have also been described in our previous study [22].

For this post hoc analysis, only patients with adequately stored blood samples were eligible for inclusion. Of the 50 patients originally randomized, two in the Theranova group (one withdrew consent and the other had insufficient samples) and four in the high-flux group (all due to insufficient samples) were excluded (Figure 1). Accordingly, 23 patients in the Theranova group and 21 patients in the high-flux group completed the 12-week study protocol and were included in the final analysis.

The primary endpoint was the PLR, calculated as the ratio of platelet count to lymphocyte count. Secondary endpoints included changes in PLR over 12 weeks relative to baseline and the reduction ratios of various inflammatory markers. This study received approval from the Institutional Review Board of Kyungpook National University Hospital (KNUH 2017–11-024; approval date: 4 January 2018) and adhered to the principles of the Declaration of Helsinki.

### 5.2. Data Collection and Analyses

Data on patient demographics, dialysis prescriptions, and laboratory findings were prospectively collected from electronic medical records at the time of study enrollment. Patients who had been receiving maintenance high-flux HD were randomized to continue high-flux HD (Fx corDiax 80 or 60; Fresenius Medical Care, Bad Homburg vor der Höhe, Germany) or to switch to HDx using an MCO dialyzer (Theranova 400; Baxter, Hechingen, Germany). All patients underwent HD with a blood flow rate of approximately 240 mL/min, a dialysate flow rate of 500 mL/min, and a session duration of about 4 h, as specified in the study protocol (Table 1).

Blood samples were collected at baseline and at 12 weeks, before and after the mid-week dialysis session. Post-dialysis blood samples were obtained using the slow-flow method [23], in which the blood pump speed is temporarily reduced to minimize recirculation and obtain an accurate post-dialysis sample. All samples were drawn into serum tubes, centrifuged for 10 min at 3000 rpm at 4 °C, and the serum was immediately frozen at −80 °C until analysis. The inflammatory markers analyzed included FGF-23, TNF-α, and IL-6. Concentrations of the inflammatory markers were measured using commercially available Enzyme-linked immunosorbent assay (ELISA) kits: FGF-23 with the Human intact FGF-23 ELISA kit (Kainos Laboratories, Tokyo, Japan), TNF-α with the Human TNF-α ELISA Kit (R&D systems, Minneapolis, MN, USA), IL-6 with the Human IL-6 HS ELISA Kit (R&D systems, Minneapolis, MN, USA). The detection ranges of respective assays were as follows: FGF-23, 3–800 pg/mL; TNF-α, 15.6–1000 pg/mL; IL-6, 0.2–10 pg/mL. Samples exceeding the upper detection limit were re-assayed after appropriate dilution. All the assays were performed according to the manufacturer’s instructions. The Bergstrom and Wehle formulas were applied to calculate the reduction ratios of these markers using pre- and post-dialysis biochemical parameters.

### 5.3. Statistical Analyses

The Kolmogorov–Smirnov test was used to assess the normality of the data. Continuous variables were reported as means ± standard deviation, and categorical variables were reported as median (interquartile range). Differences between groups were evaluated using Student’s *t*-tests or Mann–Whitney U tests for continuous variables, and Pearson’s chi-square tests or Fisher’s exact tests for categorical variables. We performed the multivariable linear regression analysis was employed to identify factors associated with changes in PLR. The generalized estimating equation models analyzed differences in PLR measurements between groups. The paired *t*-test compared the reduction ratio of inflammatory markers between the MCO and high-flux dialyzer groups. Statistical analyses were performed using SPSS version 22.0 (SPSS, Chicago, IL, USA). A *p* value less than 0.05 was considered statistically significant.

## Figures and Tables

**Table 1:** Baseline characteristics.

	Theranova (*n* = 23)	High-Flux (*n* = 21)	*p*
Age, years	62.9 ± 13.5	62.6 ± 15.6	0.95
Sex, male, *n* (%)	16 (69.6)	13 (61.9)	0.59
Body mass index, kg/m^2^	22.0 ± 2.6	22.1 ± 4.0	0.94
Dialysis vintage, months	85.8 ± 49.6	78.8 ± 47.9	0.64
Primary renal disease, *n* (%)			
Diabetes mellitus	10 (43.5)	11 (52.4)	0.56
Hypertension	3 (13.0)	3 (14.3)	>0.99
Glomerulonephritis	9 (39.1)	4 (19.0)	0.15
Others	1 (4.3)	3 (14.3)	0.34
Comorbid conditions, *n* (%)			
Diabetes	11 (47.8)	13 (61.9)	0.35
Hypertension	19 (82.6)	17 (81.0)	>0.99
Cardiovascular disease	9 (39.1)	4 (19.0)	0.15
Pre-dialysis SBP (mmHg)	145.2 ± 18.1	145.9 ± 20.3	0.91
Pre-dialysis DBP (mmHg)	68.8 ± 17.4	67.4 ± 15.2	0.77
Blood flow rate (mL/min)	246.1 ± 21.1	237.6 ± 19.7	0.18
Dialysate flow rate (mL/min)	500	500	NA
Dialysis time (min)	241.2 ± 6.2	237.0 ± 13.1	0.18
Target body weight (kg)	61.2 ± 7.7	57.3 ± 10.1	0.15
spKt/V	1.62 ± 0.21	1.67 ± 0.21	0.40
Laboratory findings			
Hemoglobin (g/dL)	10.6 ± 0.9	10.6 ± 1.2	0.87
Sodium (mEq/L)	137.0 ± 2.8	136.9 ± 2.7	0.91
Potassium (mEq/L)	4.9 ± 0.7	4.5 ± 0.7	0.09
Albumin (g/dL)	4.2 ± 0.3	4.1 ± 0.4	0.40
Calcium (mg/dL)	9.3 ± 0.7	9.0 ± 0.4	0.10
Phosphate (mg/dL)	3.9 ± 0.9	4.2 ± 0.9	0.35
Intact parathyroid hormone (pg/mL)	178.6 ± 125.9	187.2 ± 124.2	0.82

Data are presented as mean ± standard deviation or number (%). Abbreviations: SBP, systolic blood pressure; DBP, diastolic blood pressure; spKt/V, single pool Kt/V; NA, not applicable.

**Table 2:** Comparisons of the changes in inflammation-related markers in both dialysis groups.

	Baseline			12-Week		
	Theranova	High-Flux	*p*	Theranova	High-Flux	*p*
White blood cell count (×10^9^/L)	6.5 ± 1.7	6.6 ± 1.9	0.82	6.4 ± 1.5	6.9 ± 2.1	0.37
Absolute neutrophil count (×10^9^/L)	4.4 ± 1.5	4.3 ± 1.5	0.82	4.1 ± 1.3	4.7 ± 1.8	0.19
Lymphocyte count (×10^9^/L)	1.3 ± 4.3	1.4 ± 3.5	0.83	1.5 ± 5.1	1.3 ± 3.6	0.30
Platelet count (×10^9^/L)	192.3 ± 99.4	192.4 ± 51.5	1.00	179.6 ± 81.0	204.3 ± 42.3	0.22
Neutrophil-to-lymphocyte ratio	3.78 ± 2.74	3.26 ± 1.16	0.43	3.06 ± 1.17	3.64 ± 1.43	0.15
Platelet-to-lymphocyte ratio	155.4 ± 78.8	144.9 ± 31.0	0.56	125.5 ± 43.5	157.4 ± 32.4	0.01
IL-6 (pg/mL)	4.7 (2.3, 8.1)	5.4 (3.6, 8.4)	0.26	5.4 (2.7, 8.0)	5.2 (3.7, 10.8)	0.44
TNF-α (pg/mL)	17.8 ± 5.1	18.0 ± 4.9	0.89	16.1 ± 3.5	19.0 ± 5.2	0.04
FGF-23 (pg/mL)	553.9 (188.2, 2087.3)	369.8 (255.7, 763.5)	0.37	632.4 (162.9, 1635.8)	396.2 (150.2, 1008.2)	0.66

Data are presented as mean ± standard deviation or median (interquartile range). Abbreviations: IL-6, interleukin 6; TNF-α, tumor necrosis factor-alpha; FGF-23, fibroblast growth factor 23.

**Table 3:** Multivariable linear regression analysis on factors associated with the relative change in the PLR from baseline to 12 weeks.

	B	SE	β	*p*
Theranova dialyzer	−20.41	9.30	−0.32	0.04
Age	−0.07	0.30	−0.03	0.82
Female	13.68	8.73	0.20	0.13
Diabetes mellitus	1.74	8.95	0.03	0.85
Cardiovascular disease	27.83	9.56	0.40	0.01
Neutrophil percentage ratio of 12-week to baseline	72.68	44.25	0.24	0.11

Abbreviations: PLR, platelet-to-lymphocyte ratio; B, unstandardized regression coefficient; SE, standard error; β, standardized coefficient.

## Data Availability

The data that support the findings of this study are not publicly available due to their containing information that could compromise the privacy of research participants; however, these are available from the corresponding authors.

## Abbreviations

The following abbreviations are used in this manuscript:

CKD	Chronic kidney disease
DBP	Diastolic blood pressure
ELISA	Enzyme-linked immunosorbent assay
ESKD	End-stage kidney disease
FGF-23	Fibroblast growth factor 23
HD	Hemodialysis
HDx	Expanded hemodialysis
IL-6	Interleukin 6
KNUH	Kyungpook National University Hospital
MCO	Medium cut-off
NA	Not applicable
PLR	Platelet-to-lymphocyte ratio
SBP	Systolic blood pressure
spKt/V	Single pool Kt/V
TNF-α	Tumor necrosis factor-α
